# LDL receptor blockade reduces mortality in a mouse model of ischaemic stroke without improving tissue-type plasminogen activator-induced brain haemorrhage: towards pre-clinical simulation of symptomatic ICH

**DOI:** 10.1186/s12987-017-0081-2

**Published:** 2017-11-21

**Authors:** Be’eri Niego, Brad R. S. Broughton, Heidi Ho, Christopher G. Sobey, Robert L. Medcalf

**Affiliations:** 1 Molecular Neurotrauma and Haemostasis, Australian Centre for Blood Diseases, Monash University, Level 4 Burnet Building, 89 Commercial Road, Melbourne, 3004 VIC Australia; 2 Cardiovascular & Pulmonary Pharmacology Group, Biomedicine Discovery Institute, Department of Pharmacology, Monash University, Clayton, VIC Australia; 3 Vascular Biology and Immunopharmacology Group, Department of Physiology, Anatomy & Microbiology, School of Life Sciences, La Trobe University, Bundoora, VIC Australia

**Keywords:** Stroke, Tissue-type plasminogen activator, Symptomatic intracerebral haemorrhage, Blood–brain barrier, Receptor-associated protein, MCAo

## Abstract

**Background:**

Symptomatic intracerebral haemorrhage (sICH) following tissue-type plasminogen activator (rt-PA) administration is the most feared and lethal complication of thrombolytic therapy for ischaemic stroke, creating a significant obstacle for a broader uptake of this beneficial treatment. rt-PA also undermines cerebral vasculature stability in a multimodal process which involves engagement with LDL receptor-related protein 1 (LRP-1), potentially underlying the development of sICH.

**Aims and methods:**

We aimed to simulate rt-PA-induced haemorrhagic transformation (HT) in a mouse model of stroke and to assess if it drives symptomatic neurological deterioration and whether it is attenuated by LDL receptor blockade. rt-PA (10 mg/kg) or its vehicle, with or without the LDL receptor antagonist, receptor-associated protein (RAP; 2 mg/kg), were intravenously injected at reperfusion after 0.5 or 4 h of middle cerebral artery occlusion (MCAo). Albumin and haemoglobin content were measured in the perfused mouse brains 24 h post MCAo as indications of blood–brain barrier (BBB) compromise and HT, respectively.

**Results:**

rt-PA did not elevate brain albumin and haemoglobin levels in sham mice or in mice subjected to 0.5 h MCAo. In contrast, administration of rt-PA after prolonged MCAo (4 h) caused a marked increase in HT (but similar changes in brain albumin) compared to vehicle, mimicking the clinical shift from a safe to detrimental intervention. Interestingly, this HT did not correlate with functional deficit severity at 24 h, suggesting that it does not play a symptomatic role in our mouse stroke model. Co-administration of RAP with or without rt-PA reduced mortality and neurological scores but did not effectively decrease brain albumin and haemoglobin levels.

**Conclusion:**

Despite the proven causative relationship between severe HT and neurological deterioration in human stroke, rt-PA-triggered HT in mouse MCAo does not contribute to neurological deficit or simulate sICH. Model limitations, such as the long duration of occlusion required, the type of HT achieved and the timing of deficit assessment may account for this mismatch. Our results further suggest that blockade of LDL receptors improves stroke outcome irrespective of rt-PA, blood–brain barrier breakdown and HT.

## Background

Intracranial bleeding episodes, or haemorrhagic transformations (HTs), are common in ischaemic stroke [1]. They can occur spontaneously and, according to large meta-analyses, are present in 24.2% of placebo-treated and 32.5% of recombinant tissue-type plasminogen activator (rt-PA)-treated patients [2]. However, only those bleeding events which lead to worsening of neurological outcomes, so-called symptomatic intracerebral haemorrhages (sICH), have direct impact on the safety assessment of rt-PA treatment.

sICH is the most serious complication of thrombolysis with rt-PA in stroke, often resulting in devastating consequences. It occurs in ~ 6% of thrombolysed patients and carries 50% mortality rate [1, 3], causing reluctance among some practitioners to employ rt-PA-induced thrombolysis [4, 5]. Furthermore, a lack of research exploring the most appropriate management of sICH prevents the establishment of standardised guidelines for treatment of this condition [6]. There is therefore a need to identify those who are at particular risk and to develop research tools to specifically combat this emergency situation.

HTs during stroke are not uniformly presented but classified radiologically as haemorrhagic infarctions (HI-1 and -2, characterised by petechial bleeding) or parenchymal haematomas (PH-1 or -2, defined as haemorrhage occupying < 30 or > 30% of the ischaemic area with mild or significant space-occupying effect, respectively) [1, 3, 7]. While all types of HTs can be accompanied by neurological decline, it is mainly the PH-2 type that independently predicts neurological deterioration at 24 h and poor outcomes at 3 month post stroke [1, 8], particularly if it coincides with other time and neurological criteria [9].

Notably, many animal studies which assess rt-PA-related HTs do not classify the type of haemorrhage or determine whether it plays a role in functional outcome post-treatment [10–15]. Even in rodent studies that use the clinical HT classification, no correlations are performed to evaluate whether brain haemorrhage actually contributes to neurological decline [16–20]. Taken together, the nature of HTs in current pre-clinical stroke models needs further examination to allow refined simulation of the human rt-PA-related sICH.

Regardless of its outcome, for blood extravasation to occur the blood-brain barrier (BBB) must be compromised. With this in mind, considerable pre-clinical and clinical data suggests that rt-PA disrupts the BBB, potentially contributing to subsequent HT [21]. This occurs via plasmin-dependent or -independent effects of rt-PA on various cellular and acellular components of the BBB [22, 23].

Members of the low-density lipoprotein receptor (LDLR) superfamily, in particular LDLR-related protein 1 (LRP-1) [24], have long been considered to serve as t-PA receptors in the CNS [24, 25]. Many t-PA-associated BBB breakdown processes during stroke involve t-PA engagement with LRP-1, an interaction which drives upregulation of matrix metalloproteinase (MMP)-3 [13], MMP-9 [26, 27] and vascular endothelial growth factor (VEGF) in brain endothelial cells [28] and microglia [29], activation of the platelet-derived growth factor (PDGF)-CC [12] and Rho-kinase pathways [30] as well as direct cleavage of LRP-1 [31] in astrocytes and control of vascular tone [32] via stimulation of smooth-muscle cells [33].

The pan LDLR antagonist, receptor-associated protein (RAP) [34], is a small intracellular chaperone which prevents premature ligand binding to LRP-1 and also to other LDLRs such as megalin (gp330; LRP-2), the very low density lipoprotein (VLDL) receptor and the LDLR itself [34, 35]. RAP improved functional outcome and HT in rats after stroke [36] and reduced rt-PA-mediated BBB disruption [28, 31] and brain haemorrhage [13] in permanent and thrombotic mouse models of middle cerebral artery occlusion (MCAo), respectively. Yet, the efficacy of RAP against rt-PA damage in transient mechanical MCAo, which best enables focusing on off-target effects of rt-PA, has not been reported.

Here, we simulated rt-PA-triggered HT formation and examined its characteristics by appearance (HI or PH) and functional consequences in a mouse MCAo model. We further tested the potential of RAP to reduce rt-PA-induced BBB disruption and bleeding complications. Our results highlight the challenges in pre-clinical simulation of sICH and interestingly suggest that the use of RAP as a sole therapeutic agent might offer benefits in stroke despite being insufficient for effective protection of the BBB from rt-PA during thrombolysis.

## Methods

### Reagents

Human t-PA (rt-PA; Actilyse®) was purchased from Boehringer Ingelheim GmbH (Rhein, Germany) and dialysed against 0.35 M HEPES–NaOH, pH 7.4, to remove the original components of the formulation buffer [37]. Low-endotoxin human RAP was obtained from Molecular Innovations (Novi, MI, USA).

### Mouse stroke model and drug treatment

All animal procedures were undertaken in accordance with the National Health and Medical Research Council (NHMRC) “Code of Practice for the Care and Use of Animals for Experimental Purposes in Australia” and were approved by an Animal Ethics Committee of Monash University. Animal procedures also complied with the ARRIVE guidelines (Animal Research: Reporting in Vivo Experiments).

Middle Cerebral Artery occlusion (MCAo): 93 8–12 week-old C57Bl/6 male mice weighing ~ 25 g underwent MCAo (16 and 63 animals for 0.5 and 4 h occlusion periods, respectively) or sham surgery (12 animals) as described below [38]. 77 mice underwent successful experimental protocol (defined as uncomplicated surgery with successful occlusion and good recovery, complete drug treatment and satisfactory intracardial perfusion as judged by liver colour). 16 additional mice (2 shams and 14 MCAo) failed to meet these criteria and were not analysed. The overall mortality by 24 h after successful surgeries was 6.25% (1 out of 16 mice) or 34.7% (17 out of 49) in mice undergoing 0.5 or 4 h MCAo, respectively.

Focal cerebral ischemia was induced in anesthetized mice (ketamine: 80 mg/kg plus xylazine: 10 mg/kg intraperitoneally) by occlusion of the MCA using a 6.0 silicone-coated monofilament (Doccol Corporation, MA, USA). Occlusion was sustained for either 0.5 or 4 h, and the filament was then retracted to allow reperfusion. Both successful occlusion (> 70% reduction in cerebral blood flow; CBF) and reperfusion (> 90% return of CBF to pre-ischemic levels) was verified by transcranial Laser-Doppler flowmetry (PeriMed, Sweden). Head and neck wounds were stitched closed, covered with Betadine® (Sanofi, Australia) and spray dressing, and the mice were then returned to their cages after regaining consciousness. To restore blood flow in the 4 h occlusion cohort, mice were re-anesthetised 15 min before the end of the occlusion period. The wound was re-opened and the filament was retracted to allow reperfusion. Rectal temperature was monitored and maintained at 37.0 ± 0.5 °C throughout the procedure. Sham-operated mice were anesthetized and the right carotid bifurcation exposed, but no filament was inserted.

Drug treatment: rt-PA (10 mg/kg) or its HEPES vehicle were administered in both 0.5 and 4 h MCAo protocols immediately after reperfusion as a bolus injection via the tail vein (4 ml/kg; 100 µl per 25 g mouse). In selected cohorts of animals undergoing 4 h MCAo, RAP (2 mg/kg) [13, 28] was co-administered with vehicle or rt-PA in the same intravenous injection. Notably, similar studies in mice reported good response to intravenous RAP already at 1 mg/kg and only marginal improvement with dose escalation to 2 mg/kg [13, 28], suggesting that RAP at 2 mg/kg is sufficient to exert its maximal effect in our settings.

End point: 24 h post stroke mice were killed by isoflurane and then transcardially perfused with phosphate-buffered saline supplemented with 0.05 U/ml clexane using a peristaltic pump. Brains were finally removed, divided into ipsilateral and contralateral hemispheres and processed as described below.

### Mortality

**Fig. 1:**
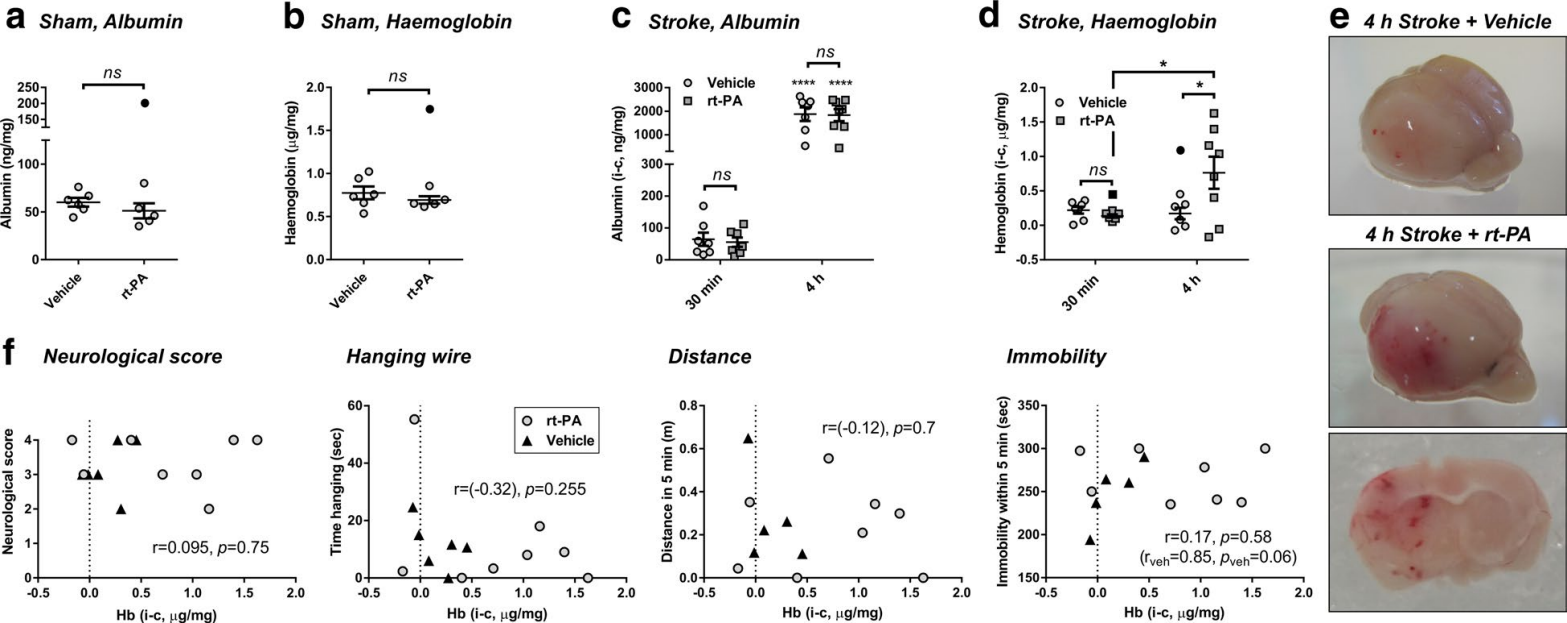
Delayed administration of rt-PA in mouse stroke induces intracerebral haemorrhage which does not correlate with neurological deficit. Human recombinant t-PA (rt-PA; 10 mg/kg) or its HEPES vehicle were administered to mice after sham operation (n = 5 or 6, respectively) or post middle cerebral artery occlusion (MCAo) for 0.5 (n = 7 each group) or 4 h (n = 8 or 7, respectively). **a**, **b** No changes in brain albumin (**a**) and haemoglobin levels (**b**) were observed between rt-PA and vehicle in sham-operated mice at 24 h after surgery. **c** Brain albumin sharply increased in the ipsilateral hemisphere 24 post 4 h MCAo compared to 0.5 h MCAo, yet no differences were detected with rt-PA compared to vehicle treatment in both time points. **d** rt-PA increased brain haemoglobin in the ipsilateral hemisphere (indicating development of intracerebral haemorrhage by 24 h) only when injected at 4 h, but not 0.5 h, post MCAo. **e** Representative images of the ipsilateral hemisphere 24 h after vehicle or rt-PA treatment post 4 h MCAo. Accumulation of blood in the stroke-affected brain and formation of multiple petechial haemorrhagic infarctions are apparent with rt-PA. **f** Correlations between brain haemoglobin levels measured in the 4 h stroke cohort (black up-pointing triangle vehicle- and white circle rt-PA-treated) and functional tests performed, including (from left to right) neurological scoring, hanging wire test and ANY-Maze recordings over 5 min of distance travelled and immobility periods. Brain haemoglobin does not correlate with any functional parameter. Data in **a**–**d** is shown as individual animals with mean ± SEM. n = 5–8 (see detailed n numbers above). Statistical analysis in **a** and **b** by student t-test; in **c** and **d** *P < 0.05, ****P < 0.0001 by 2-way ANOVA with Sidak post hoc; in **f** by Spearman correlation for neurological score or Pearson correlation for all other tests. In panels **a**–**d** Outliers are denoted in black symbols and excluded from the analysis

Mortality occurring between the time of drug treatment at reperfusion and 24 h post MCAo was reported in a targeted cohort of mice which were operated on exclusively by two experienced operators over a limited 3-month period. In this cohort, rt-PA or its HEPES vehicle were compared head to head with or without RAP. Earlier cohorts of animals treated with rt-PA or vehicle (Fig. 1) were not included in the mortality sum. 31 animals in total underwent surgery at this stage. 4 surgeries were unsuccessful and 1 animal was excluded due to severe bleeding from neck sutures, which was unrelated to drug treatment. 26 animals were therefore included in the mortality analysis. Out of 9 mortality events in this cohort, 1 mouse died 2 h after treatment (with HEPES vehicle + RAP) while all other mortalities occurred overnight (> 6 h post occlusion).

### Functional testing and neurological deficit scoring

23 h after stroke mice were scored for neurological deficit using a 5-point scoring system where 0 = normal motor function, 1 = flexion of torso and contralateral forelimb when mouse is lifted by the tail, 2 = circling to the contralateral side, but normal posture at rest, 3 = leaning to the contralateral side and 4 = no spontaneous motor activity [38]. Mice were then placed for 5 min in a parallel rod floor apparatus of an ANY-maze (Stoelting, IL, USA), an automated behaviour tracking system which records spontaneous movements on a grid of rods and extracts parameters such as distance, speed, immobile episodes, circling behaviour and foot faults. Finally, a grip strength hanging wire test was performed as described [38], averaging three trials with 5 min rest intervals. Scores ranged from 0 for animals which fell immediately to a maximum of 180 s, typically achieved by sham-operated mice.

### Intracerebral bleeding assessment

Extravasation of red blood cells into brain parenchyma, indicative of HT following severe malfunction of cerebral blood vessels, was measured in perfused ipsilateral and contralateral brain hemispheres. Hemispheres were first weighed, then homogenised to 300 mg/ml (wet weight) in PBS + 1% Triton X-100 (v/v) and snap-frozen on dry ice. Lysates were thawed before use and spun down at 16,100 g for 10 min. The supernatant was further diluted to 150 mg/ml (wet weight) in equal volume of milli-Q water. Haemoglobin (Hb) concentration in brain supernatants was assessed spectrophotometrically by the QuantiChrom™ Hemoglobin Assay Kit (BioAssay Systems, CA, USA) as per the manufacturer’s instructions, incubating samples with the reagent for 15 min [10] before reading absorbance at 405 nm on a micro-plate reader (VICTOR II, Perkin Elmer, Australia). Hb content in each hemisphere was calculated as µg per mg wet brain tissue using the formula: Hb (µg/mg wet weight) = Hb (µg/ml)/300 mg/ml. In mice which underwent stroke surgery (but not in shams), Hb level obtained in the contralateral hemisphere was subtracted from the ipsilateral hemisphere to account for perfusion efficiency under the assumption that both hemispheres are perfused to a similar degree and that the contralateral value represents the amount of intravascular blood remaining in the brain post perfusion. Importantly, no significant differences were noted in the Hb content of the contralateral hemispheres between vehicle- and rt-PA-treated animals (not shown).

### Evaluation of brain albumin content as a measure of blood–brain barrier disruption

Extravascular albumin is scarcely present in brain parenchyma [39] and its detection at greater levels in perfused brains indicates an increase in BBB permeability. To assess the degree of BBB compromise, brain lysates at 300 mg/ml (wet weight) were thawed and spun down as stipulated above. Supernatants were diluted further in PBS so the final sample concentration in the ELISA plate was 25 or 250 µg/ml (wet weight) for the ipsilateral or contralateral hemisphere, respectively. Brains of sham-operated mice were processed as the contralateral hemispheres. We then quantitated brain albumin concentration in the supernatant by ELISA using a commercial kit (Mouse Albumin ELISA Quantitation Set #E90-134; Bethyl Laboratories, TX, USA) according to the manufacturer’s instructions [40]. Brain albumin content in the perfused brain was calculated according to the formula: Albumin (ng/mg wet weight) = Albumin (ng/ml)/300 mg/ml. As stated above for the analysis of Hb and under the same assumptions, albumin levels obtained in the contralateral hemispheres were subtracted from the ipsilateral hemispheres in stroke mice to account for perfusion efficiency.

### Statistical analysis

**Fig. 2:**
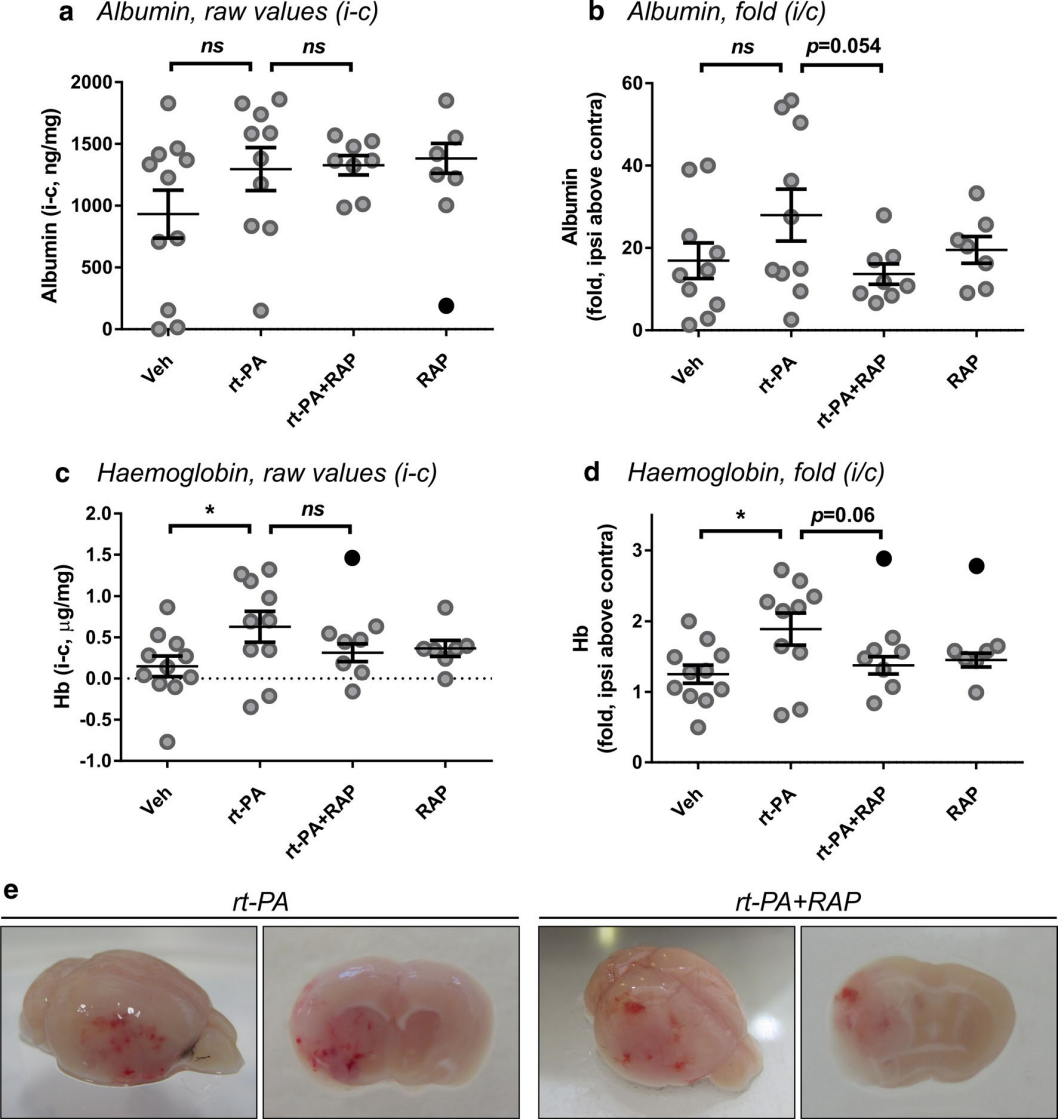
Receptor-associated protein (RAP) does not significantly attenuate rt-PA-induced BBB disruption and formation of intracerebral haemorrhage. Receptor-associated protein (2 mg/kg) was co-administered with rt-PA (10 mg/kg) post 4 h middle cerebral artery occlusion (MCAo) and blood components were measured in the perfused brain 20 h later. **a** Albumin values in the ipsilateral hemisphere (after subtraction of the contralateral values to correct for perfusion efficiency) and **b** fold analysis of brain albumin (ipsilateral above contralateral, accounting for each animal’s own baseline). Administration of RAP together with rt-PA does not reduce albumin levels compared to rt-PA alone (**a**) but a trend for reduced albumin with RAP is apparent by fold (**b**). **c**, **d** Brain haemoglobin is increased with rt-PA compared to vehicle both in the raw analysis (ipsilateral minus contralateral; **c**) or the relative analysis (fold; ipsilateral above contralateral; **d**). While a trend emerges, addition of RAP with rt-PA does not significantly attenuate blood levels in the brain. **e** Representative images of brain tissue 24 h after rt-PA or rt-PA + RAP treatment post 4 h MCAo. Significant intraparenchymal hematomas can still be observed after RAP treatment. Data is shown as individual animals with mean ± SEM. n = 11 for vehicle, 10 for rt-PA, 8 for rt-PA + RAP and 7 for RAP. One-way ANOVA with Sidak post hoc analysis of selected groups. Outliers are denoted in black symbols and excluded from the analysis

**Fig. 3:**
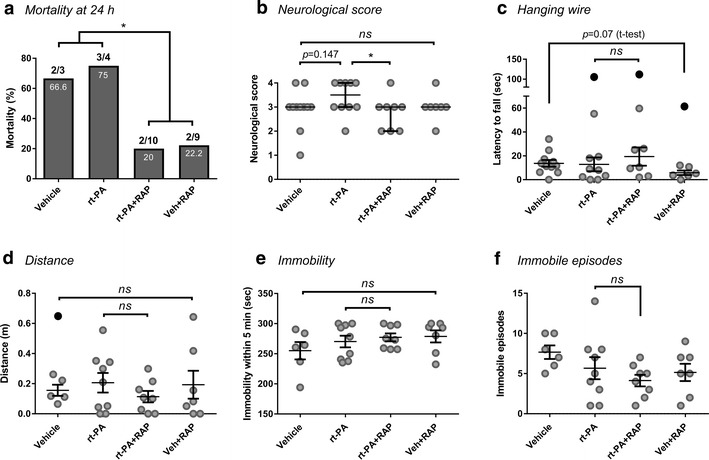
Receptor-associated protein (RAP) reduces mortality and neurological score but does not improve other functional parameters. Mortality rates (**a**) and neurological deficit assessment (**b**–**f**) 20 after 4 h middle cerebral artery occlusion following treatment with HEPES vehicle, rt-PA (10 mg/kg), RAP (2 mg/kg) or their combination. RAP reduced mortality with or without rt-PA (**a**) and improved neurological score when co-administered with rt-PA (**b**). In other functional tests, such as hanging wire (**c**) and ANY-Maze recording over 5 min of total distance travelled (**d**), total time immobile (**e**) and the number of immobile episodes (**f**), RAP treatment did not offer any functional benefit. Data is shown as individual animals with median + IQR (**b**) or mean ± SEM (**c**–**f**). In **a** black and white annotations above each column stipulate fatalities out of total animal number and percentage of death in each group, respectively. In **b**, **c** n = 11 for vehicle, 10 for rt-PA, 8 for rt-PA + RAP and 7 for RAP. In **d**–**f** n = 6 for vehicle, 9 for rt-PA, 8 for rt-PA + RAP and 7 for RAP. In **a** *P < 0.05 by Log-rank (Mantel-Cox) test; in **b** *P < 0.05 by Kruskal–Wallis ANOVA with Dunn’s multiple comparisons test of selected groups; in **c**–**f** ordinary one-way ANOVA with Sidak’s post hoc of selected groups unless t-test is specified. Outliers are denoted in black symbols and excluded from the analysis

Statistical analysis was performed using GraphPad Prism 6 software. Outliers were first determined in all data sets by Grubbs’ test with α ≤ 0.1 and excluded from the analysis. No more than one outlier was identified in any group (see Figs. 1, 2, 3) and its exclusion did not affect the observation. Mortality was analysed by Log-rank (Mantel-Cox) test. Differences between three or more groups were analysed by ordinary one- or two-way ANOVA with Sidak’s post hoc analysis (for parametric data) or by Kruskal–Wallis ANOVA with Dunn’s multiple comparisons test (for non-parametric data). Differences between two groups were determined using two-tailed unpaired student *t* test. Correlations were assessed by Spearman correlation for non-parametric data or by Pearson correlation for parametric data. Probability values under 0.05 were considered significant.

## Results

rt-PA administration is approved within 4.5 h from stroke onset. Beyond this time window, its risks, primarily for development of sICH and/or oedema following breach of the BBB, outweigh the potential clinical benefits. In an attempt to mimic these occurrences in the mouse we first characterised the degree of BBB disruption (extravasation of albumin and haemoglobin into the brain) and functional deficit in response to rt-PA under sham conditions and in our MCAo stroke model. In line with other pre-clinical reports [12, 26], intravenous rt-PA (10 mg/kg) administered to uninjured animals did not induce albumin (Fig. 1a) or blood (Fig. 1b) accumulation in the mouse brain 24 h post treatment, suggesting that rt-PA does not affect the mouse BBB under resting conditions (note that no subtraction of the contralateral from the ipsilateral value was performed in shams). In contrast, transient stroke as short as 0.5 h followed by intravenous treatment at reperfusion disrupted the BBB and increased albumin content in the ipsilateral hemisphere by 24 h post MCAo (observed as positive values after subtraction of the contralateral hemisphere) in both vehicle and rt-PA-treated mice, an effect which was dramatically amplified by 29- and 33.1-fold, respectively, when stroke duration was extended to 4 h (*p* < 0.0001, Fig. 1c). Notably, albumin accumulation at 24 h did not significantly differ between rt-PA and vehicle in both 0.5 and 4 h stroke periods, indicating that a similar degree of BBB permeation to plasma proteins developed with or without the thrombolytic (Fig. 1c) (see “Discussion”). Brain haemoglobin levels, however, were significantly increased by 24 h in mice receiving rt-PA at 4 h, but not at 0.5 h post MCAo or in vehicle-treated animals (*p* < 0.05, Fig. 1d), suggestive of development of significant HTs only during delayed use of rt-PA. Indeed, macroscopic examination of perfused brains 24 after 4 h MCAo revealed no bleeding or sporadic small petechiae in brains of vehicle-treated animals but extended bleeding with multiple sites of haematomas in most rt-PA-treated mice (Fig. 1e). Hence, our stroke model successfully simulated the time-dependent shift of rt-PA from a safe to unsafe intervention as judged by formation of substantial HT.

We then examined whether these extensive bleeding complications were contributing to functional outcome; in other words, if they could be considered ‘symptomatic’. Noticeably, none of our functional assessments performed 24 h after MCAo (neurological scoring, hanging wire and ANY-maze tests) correlated with the brain haemoglobin levels measured at this time point, irrespective of vehicle or rt-PA (Fig. 1f). This analysis shows that HTs associated with rt-PA in these experimental stroke settings are not predictive of deficit severity and cannot be considered symptomatic. Importantly, autopsies performed on animals which died before the 24 h end-point (transcardially-perfused where possible) did not reveal consistent HTs, including in rt-PA-treated animals (not shown), suggesting that HT was not playing a major role in their functional deterioration and early death.

LDLR blockade by RAP may provide a unifying solution to a number of rt-PA modes of action which lead to BBB disruption and HT during thrombolysis [22, 23]. We therefore tested the effects of RAP against rt-PA-induced BBB breakdown and formation of HT as well as mortality and functional outcome in our 4 h transient thread occlusion protocol, which to the best of our knowledge has not been attempted before. As observed in our initial characterisation (Fig. 1), rt-PA treatment induced a similar increase in albumin extravasation as seen for vehicle-treated mice (Fig. 2a, b) but caused a significant upsurge in the brain haemoglobin content measured 24 h after stroke (*p* < 0.05, Fig. 2c, d). Administration of RAP (2 mg/kg) at reperfusion with or without rt-PA did not significantly decrease total brain albumin levels (Fig. 2a), yet a trend for reduction of total brain haemoglobin was observed (*p* = 0.18, Fig. 2c). Normalisation of the ipsilateral value to the contralateral baseline for each mouse (i.e. fold increase analysis) revealed near-significant reductions in cerebral albumin (*p* = 0.054, Fig. 2b) and haemoglobin content (*p* = 0.06, Fig. 2d) by RAP. Indeed, focal haematomas and diffuse bleeding across the infarct could often be observed in rt-PA + RAP-treated brains 24 h post MCAo, as seen with rt-PA alone albeit to a milder extent (Fig. 2e), in line with the biochemical findings. As mentioned above, our autopsies of mice which died before the 24 h end-point and were not included in the biochemical analysis did not establish a contributory relationship between HT and mortality, reducing the possibility of underestimation of the RAP effect on the BBB. Overall, these data suggest that RAP does not protect the BBB from rt-PA to an extent where blood cells and blood protein extravasation can be effectively inhibited, but may still harbour a partial capacity to attenuate rt-PA-induced development of HT.

Interestingly, despite its weak activity on the BBB, RAP treatment with or without rt-PA resulted in a marked decrease in mortality within 24 h from stroke onset (from 71.4% overall mortality in vehicle and rt-PA groups to 21% in RAP-treated groups, *p* < 0.05, Fig. 3a). RAP administration also resulted in a functional benefit since rt-PA + RAP-treated animals received on average significantly lower neurological scores than mice treated with rt-PA alone (*p* < 0.05, Fig. 3b). A strong trend for improved grip strength was further demonstrated with RAP in vehicle-treated (*p* = 0.07 by t-test, Fig. 3c), but not in rt-PA-treated animals. Notably, none of the mobility parameters assessed by ANY-maze at 24 h were influenced by RAP (Fig. 3d–f). Taken together, these observations suggest that LDLR blockade during severe stroke could offer fundamental benefits such as improved (short-term) survival and attenuation of deficit, which may not be related to rt-PA, BBB breakdown or brain haemorrhage.

## Discussion

Because severe intracranial haemorrhage is the main complication limiting thrombolysis with rt-PA in stroke [1, 3, 6], we attempted to simulate rt-PA-driven HT in a mouse stroke model and to test whether LDLR blockade could protect against its occurrence. However, since only symptomatic bleeding episodes (sICH) are pertinent for the safety assessment of rt-PA, we further evaluated if HTs obtained in the mouse actually carry the functional and structural characteristics of the clinical condition, such as neurological deterioration and development of parenchymal haematoma type-2 (PH-2), respectively [8]. This distinction is rarely made in rodent stroke studies but may be important for improvement of research efforts.

In line with pre-clinical reports by others [17, 18, 41], rt-PA caused substantial HTs in our study by 24 h post stroke, but only when given 4 h after stroke initiation. Rodent studies therefore mimic the basic clinical observation showing lower incidence of severe intracranial bleeding at onset to treatment (OTT) ≤ 90 min and a shift towards higher risk of dangerous bleeding outcome at later OTTs [2], which usually occurs within 24–36 h [6]. Interestingly, despite the increase in rt-PA-mediated HT after prolonged occlusion, we saw no differences between rt-PA- and vehicle-treated groups in albumin extravasation into the brain within 24 h regardless of stroke duration. These observations provide valuable insight into the most significant activities of rt-PA on the BBB, as they presumably stem from differential opening of the BBB to varying degrees, allowing passage of different sized particles depending on the severity of disruption. Albumin (and other plasma proteins) can penetrate the brain paracellularly through damaged tight junctions or transcellularly via transcytosis [39], while passage of red blood cells into the brain requires complete capillary disintegration. Hence, it seems that the long occlusion duration (4 h) is sufficient to substantially compromise the BBB for extravasation of plasma proteins regardless of rt-PA (or, that the effect of prolonged MCAo on albumin extravasation is robust enough to reach saturation within 24 h—the time of our evaluation—and mask early acceleration in this process by rt-PA). However, only rt-PA seems to drive the vessel further towards final catastrophic breakdown, permitting the passage of whole blood into the brain. Such vessel disintegration must involve mechanical rupture and total loss of key structural elements of brain capillaries like the lumen-forming endothelial cells and the supportive basement membrane; accordingly, robust rt-PA-associated activities such as plasmin- and MMP-dependent proteolytic degradation of basal lamina and tight junctions [22], worsening of cerebral inflammation by reduction of regulatory T-cells [42] and recruitment of other immune cells [21] as well as changes to blood pressure and vascular tone [32] should logically become therapeutic candidates in the context of sICH prevention after thrombolysis.

Despite successful modelling of the temporal bleeding formation which occurs in humans, HTs developing post-rt-PA in the mouse did not resemble clinical sICH in their appearance and functional consequence. These bleeding events, while substantial, appeared more as scattered (HI-1) or more confluent yet heterogeneous petechiae (HI-2) rather than parenchymal hematomas, characterised by a homogenous and dense haemorrhage creating a mass effect and occupying a large area of the infarct [8]. Functionally, no cause-effect relationship could be established between HT and deficit severity. Few possible contributors may account for this outcome, including a lack of sensitivity of the functional assessment methods, the absence of longitudinal HT and deficit monitoring throughout the first 24 h to identify earlier associations (before mortality occurs) or the need to administer rt-PA by infusion rather than a bolus (the latter described in rodent studies with similar outcomes [16, 17]). Nevertheless, as vehicle-treated mice generally suffered from high mortality similar to rt-PA-treated animals (Fig. 3) and displayed severe deficits 24 post 4 h MCAo (Figs. 1, 3), a lead explanation is a ‘ceiling’ effect, referring to such intense brain damage developing in the mouse as a result of complete MCA shutdown over many hours (required, however, to simulate rt-PA-dependent HT), that it cannot be modified any further when significant haemorrhage occurs [9]. This notion is highly plausible, since a typical infarct size achieved in mice by the suture MCAo model is approximately an order of magnitude larger than the infarct produced by an average human stroke (by percentage of the hemisphere affected). Equivalent massive infarcts in humans are usually accompanied by substantial oedema, progressive infarct expansion, brain herniation or pan-hemispheric destruction, with minimal functional recovery [43]. A prolonged transient mouse stroke might therefore be closer in outcome to a malignant human stroke, where a devastating outcome develops with or without HT [43]. Taken together, our study highlights substantial limitations in pre-clinical modelling of rt-PA-triggered sICH by the popular suture model of transient MCAo in mice. A more suitable protocol to study this phenomena in rodents could consist of transient occlusion of a smaller brain territory for longer periods of time (e.g. 6–9 h) before rt-PA treatment, accompanied by periodic magnetic resonance imaging (MRI). Such approach may reduce baseline deficit but allow the occurrence of substantial bleeding in severely-impaired blood vessels and their progressive monitoring. Worsening of neurological symptoms as HT occurs should be incorporated in models specifically dealing with sICH and its prevention.

A second interesting finding of our study was that LDLR blockade with the pan LDLR antagonist RAP had only a weak capacity to influence BBB-related outcomes of rt-PA treatment, yet it offered marked improvement in short-term survival and more limited attenuation of deficit severity. One might further speculate that RAP-mediated improvements in neurological function is in fact underestimated, since only animals undergoing 4 h MCAo which survived up to 24 h post-occlusion were assessed; earlier functional assessment, before mortality occurred, would have included critically ill animals and may have resulted in better correlation between function and mortality.

Regarding the BBB, while we indeed noticed a non-significant trend of decreased brain albumin with RAP, this observation overall lies in contrast to other studies demonstrating reduction of blood and blood protein extravasation into the brain by intravenous [13, 28] or intracerebroventricular [31, 36] administration of RAP, with or without rt-PA treatment. While differences in stroke models most likely contribute to these discrepancies (i.e. rat [36] vs. mice, thrombotic [13, 36] vs. mechanical or permanent [28, 31] vs. transient), our results can be logically explained by the multifaceted nature of rt-PA actions, some of which are LDLR-independent. In particular, while most plasminogen-independent pathways for rt-PA-induced BBB breakdown identify LRP-1 as a rt-PA receptor (see “Background”) [22, 23], more recently-discovered, plasmin-dependent cascades have partial or no reliance on LDLRs, including rt-PA/plasmin-driven activation of plasma kallikrein [11], bradykinin [44], monocyte chemoattractant protein (MCP)-1 [45] and Rho-kinase 2 [46]. The numerous mechanistic avenues by which rt-PA disrupts the BBB during thrombolysis implies that a sole therapeutic agent will probably be unable to achieve sufficient barrier protection. Instead, combination therapy of substances that address multiple pathways simultaneously, for example inhibitors of cell-surface receptors (Imatinib [12], RAP [13, 28], Icatibant [44]) together with plasma and intracellular enzymes antagonists (GM6001 [20], BPCCB [11] and fasudil/KD025 [30, 46], respectively) should be considered, similar to growing approaches in cancer therapy.

Importantly, rt-PA signalling via LDLRs, in particular LRP-1, also plays a key role in neuronal calcium haemostasis via regulation of the N-methyl-d-aspartate receptor function [37, 47], linking rt-PA and endogenous t-PA to pathological brain processes such as excitotoxicity and neurotoxicity [47]. Hence, even in the absence of efficient protection of the BBB, RAP might still reduce neuronal damage and infarct size, as already shown in rats [36]. Together with other effects of LDLR blockade, such as a decrease in t-PA-induced brain MMP-9 levels [29], the net consequence of RAP treatment could be a reduction in brain damage to a level where animal survival and gross neurological deficits improve, in line with our observations.

## Conclusions

In summary, we have shown here that pre-clinical modelling of rt-PA-triggered sICH during stroke only partially correlates with the clinical event, most likely owing to a mismatch between the duration of stroke and severity of outcome between humans and mice. A therapeutic strategy based on a single agent, such as LDLR blockade by RAP, has only a limited chance of success in protecting the barrier due to the diversity of mechanisms employed by rt-PA to disrupt the BBB. RAP, however, should be included since its protective action in the brain may span more than just the BBB. Future studies aiming to reduce the risk of sICH during thrombolysis will benefit from closer simulation of sICH features and from incorporation of a combination therapy to increase the relevance and translation potential of the finding.

## Abbreviations

BBB: blood–brain barrier
HI: haemorrhagic infarctions
HT: haemorrhagic transformation
LRP-1: low-density lipoprotein receptor-related protein 1
MCAo: middle cerebral artery occlusion
MMP: matrix metalloproteinase
PH: parenchymal haematoma
sICH: symptomatic intra-cerebral haemorrhage
RAP: receptor-associated protein
rt-PA: recombinant tissue-type plasminogen activator
